# Psycho-emotional distress in children and adolescents in relation to COVID-19 confinement and pandemic: A systematized review: Author lists

**DOI:** 10.1186/s13052-023-01450-7

**Published:** 2023-04-15

**Authors:** Mª Teresa García-Rodríguez, Iria Juanatey-Rodríguez, Rocío Seijo-Bestilleiro, Cristina González-Martin

**Affiliations:** 1 RN, PhD, Rheumatology and Public Health Research Group, Research in nursing and health care, Institute of Biomedical Research of A Coruña (INIBIC), Complexo Hospitalario Universitario de A Coruña (CHUAC), A Coruña, Spain; 2 RN, Complexo Hospitalario Universitario de A Coruña (CHUAC), A Coruña, Spain; 3 Public Health Research Group, Research in nursing and health care, Institute of Biomedical Research of A Coruña (INIBIC), Complexo Hospitalario Universitario de A Coruña (CHUAC), A Coruña, Spain

**Keywords:** Mental health, Adolescents, Children, Quarantine, COVID-19, Systematized review

## Abstract

A stressor such as a pandemic is a trigger to focus on the study of the psycho-emotional impact on children and adolescents from a nursing care perspective. The aim of this systematized review was to know the impact of the lockdown and COVID-19 pandemic on children (from 2 to 12 years) and adolescent (from 13 to 18 years) in relation to daily routines, as well as the most frequent psycho-emotional manifestations. The research was carried out following PRISMA guidelines and was performed using several databases (PubMed, Scopus and Cochrane). The search was focused on children and adolescent population between 2020 and 2021.The literature search was from November to December 2021. Sixteen articles were used to perform the systematized review. A disruption in daily routines and an increase in psycho-emotional manifestations have been observed in young people, which is understood as a worsening of their psycho-emotional integrity. Higher levels of anxiety and depression in adolescents and hyperactivity and dependence disorders in the children have also been reported. In conclusion, children and adolescents have been affected in the psycho-emotional sphere in the same way as adults, therefore, it is necessary to know the presenting characteristics of this group of people in order to be able to establish an effective nursing approach and help preserve the mental integrity, as well as promote resilience.

## Introduction

COVID-19 is the designation given to a variant of the coronavirus family that first appeared in the city of Wuhan, China, in December 2019. Due to its ability to spread rapidly, its transmission to the rest of the world was inevitable, leading to the declaration of a global pandemic health emergency on March 11, 2020 by the World Health Organization. This required exceptional measures to curb its spread and contagion among the population, implementing restrictive measures such as limiting the free movement of people and house confinement, among others [1].

This situation caused physical and psychological alterations in different population groups, including young people. The young population (children and adolescents) are particularly vulnerable and susceptible due to the physical, cognitive, emotional and social changes inherent to the stages of development should be taken into account.

Thus, in 2009, the Flash Eurobarometer showed the results of a study conducted in 27 countries of the European Union, with children aged 6 to 17 years. This study collected parents’ perceptions of their children’s mental health in the last week and found that the European average for emotional well-being was 74%. They also observed that 18% of parents re-ported that their children felt sad quite often and although 56% reported that their children did not feel lonely, 8% reported that they felt lonely quite often. Regarding the relationship with peers, 7% of the parents felt that they rarely had fun with friends and 2% said they never had fun. In addition, while the majority of parents felt that their children were paying attention in school, 8% reported a lack of attention in school. Finally, results from different countries in which the study was conducted ( i.e. Spain, Sweden, Finland, Luxembourg and the Netherlands) indicated lower scores in the dimensions of mood and emotions [2].

Therefore, it is essential to evaluate the impact that this confinement has had on young population and on their ability to adapt, knowing that the restrictions implied that the development of interpersonal ties, interaction with the outside world and educational processes were carried out through screens and passive observation from their homes [3].

The first published studies on psychopathological state and emotional well-being during the period of isolation were carried out focusing on the adult population, who was also influenced by added stressors such as loss of employment or income. Thus, the impact that such measures had on this group began to be studied, and an increase in the levels of anxiety and depression in the population was confirmed, especially in the female population compared to the male population [4]. After these conclusions, the evaluation of the different groups began to be considered. Due to the differences in the psycho-emotional spheres observed in the different stages of life, articles began to be published on the psychopathology and impact on the lives of children and adolescents during the isolation period. A study carried out on Chinese children in COVID-19 confinement showed an increase in anxious and depressive symptomatology [5]. Although it is not possible to apply these findings in their entirety to the European child population due to sociocultural differences, it allows us to approach the idea of the impact and psychological repercussions not only on adults, but also on the younger population.

The need to carry out this systematic literature review is due to the fact that both children and adolescents are a vulnerable group because they are in a stage of physical and mental development. This vulnerability may be increased by other factors such as family environment, socioeconomic status or educational level. Therefore, it is necessary to know how confinement has affected the psychoemotional level in order to develop prevention, care and follow-up strategies for this population, as well as to design action plans to minimize the impact of the psychoemotional alterations that may occur. Therefore, the objective of this review was to determine the psychoemotional alterations of children and adolescents during the period of covid 19 confinement and pandemic. In addition, we also wanted to know if there were differences in the psychoemotional impact depending on the country of origin and what were the risk factors or strategies to promote resilience in this population.

## Methods

### Strategy for data synthesis

This review was written following the Preferred Reporting Items for Systematic Reviews and Meta Analyses (PRISMA) protocol for systematic reviews [6]. The PICO (Patients, Intervention, Control and Outcomes) protocol was applied and the research questions were: What is the psycho-emotional impact on children and adolescents in relation to confinement and the COVID-19 pandemic? Moreover, are there differences in the psycho-emotional impact on children and adolescents depending on their country of origin?

### Search strategy and selection criteria

The search was focused on the children (from 2 to 12 years old) and adolescent (from 13 to 18 years old) population during the period of confinement by COVID 19. The outcomes were categorized as: Lifestyle changes and psycho-emotional manifestations: general results, Psycho-emotional manifestations according to age differentiation, Effects of confinement from a cross-cultural approach, and strategies for building resilience: protective factors of the emotional symptoms. We set a timeframe of research published since 2020 to 2021. The inclusion criteria were: (a) research reports about the psycho-emotional impact of confinement in Covid 19 on the child and adolescent population (b) written in English or Spanish and (c) published between 2020 and 2021.

The exclusion criteria were:

- Review articles, papers, and letters.
- Research articles that study psychoemotional alterations in populations of different age ranges and do not specify the results obtained in the child and adolescent population, giving the results at a general level.
- Those articles that assess psychoemotional alterations in children and adolescent with some underlying pathology (disabled, autistic…).

Two nurse researchers identified the Mesh terms and used the keywords to develop a rigorous search strategy in different databases (PubMed, Scopus and Cochrane) (Table 1). The search of literature was from November to December 2021.

**Table 1:** Keywords and limits used to the search strategy

Keywords	Limits
mental health,	Humans
adolescent,	Child:6–12 years
,	Child Preschool:2–5 years
children,	Adolecent:13–18 years
quarantine,	English, Spanish
,	
Covid-19	

### Data extraction and quality assessment

The initial articles selection process was carried out according to the exclusion and inclusion criteria, followed by reading the title and / or abstract. If the title responded to the subject of the review, the abstract was retrieved for reading, and if it was considered relevant, the full text was accessed. Three researchers independently, screened the titles and abstracts of the studies found to identify those that met the inclusion criteria. Then, the articles that were not discarded were read in full text and assessed for their election. Disagreements about eligibility of studies were solved through discussion and by a fourth reviewer. To assess the quality of the articles, the scientific level of evidence designed by Agency for Healthcare Research Quality (AHRQ) [7] was used due to its simplicity and clarity. A data extraction sheet was developed and the authors extracted the data. Differences were discussed face to face and, when there was consensus, the data were included. The variables were taken into account were: author, country, year of publication, level of evidence, research design, resilience-building strategies, age range of the population, psycho-emotional and behavioral manifestations, changes in routine and study objectives. The data were synthesized and analyzed by the review authors, and discrepancies were solved by consensus. The results were written as a descriptive narrative synthesis and tables were made to collect the variables taken into account.

## Results

### Selection and characteristics of sources of evidence

**Fig. 1:**
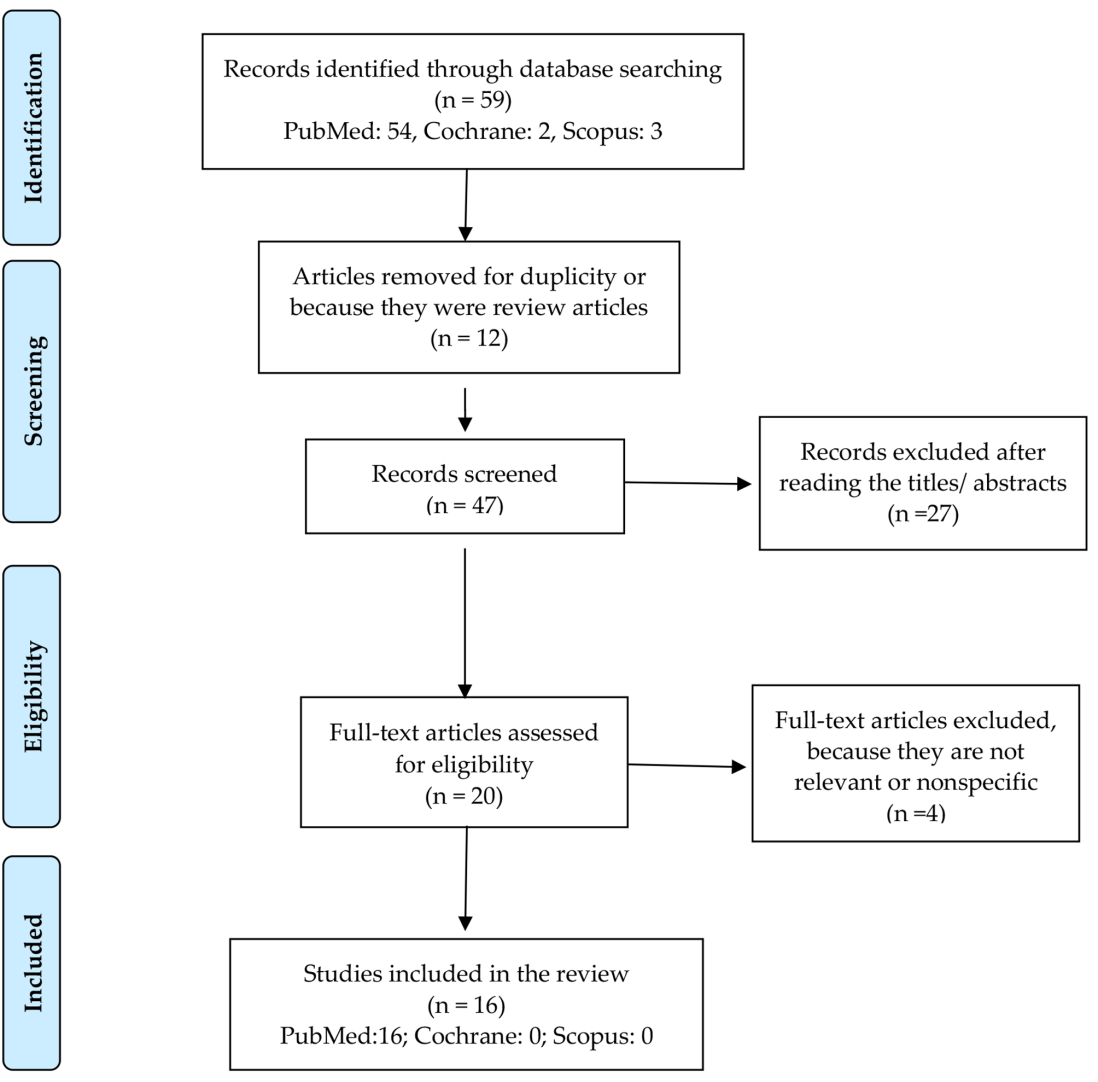
PRISMA Flow Diagram

After the bibliographic search, 59 articles were found. Twelve were removed for duplicity or because they were review articles. The remaining 47 articles were assessed by the authors in a primary review based on reading the titles and abstracts, discarding 27 articles because they did not address the issue of interest or met any of the exclusion criteria. The authors read the full text of 20 articles and 4 were excluded because they did not focus on the objective of the study due to being nonspecific or minimally relevant. A total of 16 articles were included in the systematized review for this study (Fig. 1).

Of the sixteen studies included in the review, the 37.5% were of Spanish origin [8–13] and the 12.5% were cross-cultural articles originating in Spain, Portugal and Italy simultaneously [14, 15]. The origins of the remaining articles were China[16], Australia [17], Italy [18, 19], England [20], Jordan [21],Palestinian[22],Switzerland[23]. Likewise, the cross-sectional study is the most representative of the total number of articles (81.25%) [8–10, 12, 14–16, 18–23], followed by longitudinal studies (12.5%) [13, 17] and a case control study [11]. Regarding the levels of evidence, IIa [8–10, 12–23] and III [11] according to AHRQ stand out (Table 2) (Table 3).

**Table 2:** General results

Author (Year)	Aged	Resilience strategies	Psycho-emotional manifestations/Behavior	Changes in routine	Aims
Gómez-Becerra [9] (2020)	Children and adolescent aged 3 to 18	NA	In children, predominance of emotional symptoms, hyperactivity and social behaviors. In adolescents, predominance of behavioral problems, peer relationship problems and higher scores on fear of disease transmission.	NA	To analyze the relationship between fear of illness and the development of psycho-emotional symptoms, as well as to assess the evolution of emotional, behavioral, social and fear-related problems during confinement.
Liang [18] (2020)	Children aged 6 to 12	Task-oriented, emotion-oriented and avoidance-oriented strategies, with acceptance being the most commonly used coping strategy.	Among the most common psychological responses were worry, feelings of loneliness, fear, sadness, concentration difficulties or irritability.	NA	To determine the psychological responses and coping strategies in north and center area of Italy
Erades [10] (2020)	Children aged 3 to 12	Increase physical activity time and decrease the use of screens.	Increases in emotional manifestations was observed, followed by sleep and behavioral problems, relating the increase to the progress of confinement.	Decreased physical activity and increased use of screens.	To evaluate emotional and behavioral reaction and sleep patterns during confinement. Identify adaptive variables and analyze the relationship between parental variables and manifestations in the children.
Orgilés M [11] (2020)	Children aged 6 to 12	Implementation of support programs and development of effective coping strategies in stressful situations.	Children who had not received SSL program had higher scores on anxiety, sleep, cognitive and mood disorders, as well as on emotion-based and attention-seeking coping strategies, while the group that had received SSL program had lower scores on more adaptive coping styles.	NA	To compare coping styles and psychological impact between children who had received SSL program and those who had not.
Morgül E [20] (2020)	Children aged 6 to 12	NA	Increased emotional and behavioral symptoms (irritability, frustration, boredom, demand for attention, increased intrafamily arguments, sadness...). The greater the distress in adults, the greater the impact on young people.	More screen time and less time for sleep and physical activity.	To assess emotional state and daily activities during confinement, as well as the association between parental mental health and the impact on children.
Tamarit A [12] (2020)	Adolescent aged 13 to 17	NA	Girls are more likely to have higher scores for anxiety, depression and stress than boys. Volunteering, housing without open spaces, increased time spent in confinement, and excessive searches for information on COVID-19 increase levels of depression, stress, and anxiety.	NA	To evaluate which sociodemographic variables are predictors of stress, depression and anxiety in adolescence, also taking into account gender factors.
Ezpeleta L [13]	Adolescent mean age 13.9	NA	The most significantly affected areas were behavioral problems, peer relationships and social behaviors. Likewise, there are hyperactivity problems mostly related to school workload. On the other hand, intrafamily arguments, stress and sleep problems increased.	Disruption of routine and social isolation. Worsening of intrafamily relationship, maintenance of homework and greater involvement in family activities, increased screen time and decreased physical activity.	To study the living conditions of adolescents during confinement and their correlation with their psychological state.
Pizarro-Ruiz [8] (2021)	Children and adolescent aged 8 to 18	NA	Children show alterations in the affective area with more rebellious behaviors, while adolescents show more anxiety, depression, emotional regulation problems or low self-esteem.	NA	Assessing the effects of confinement on Spanish children and adolescents
Tang [16] (2021)	Children and adolescent aged 6 to 17	Open communication between parents and children	Senior secondary school students had higher levels of depression, stress and anxiety than younger students. While primary and junior secondary students were the least satisfied with life.	NA	To assess the levels of depression, anxiety, stress and life satisfaction of young people and correlate them with parent-child dialogue.
Orgilés M [14] (2021)	Children and adolescent aged 3 to 18	NA	Spanish and Italian youths showed greater manifestations of anxiety, stress and depression than Portuguese youths.	Household confinement with permission for short daytime walks, with the exception of Portugal, which did not decree mandatory confinement, and closure of educational institutions.	To evaluate anxious-depressive symptoms in order to know which variables are related to worse well-being during confinement.
Francisco R [15] (2020)	Children and adolescent aged 3 to 18	NA	Behavioral disturbances, anxiety and mood disturbances were more frequent in Spanish and Portuguese youths than in Italians.	Increased consumption of screens and new technologies, decreased physical activity and increased sleep time.	To describe and establish a comparison between psychological and behavioral symptomatology in relation to confinement and COVID-19.
Magson [17] (2021)	Adolescent aged 13 to 16	NA	Increase depressive and anxiety symptoms in adolescents, with a decrease in life satisfaction. These symptoms are more pronounced in girls. The adolescents with more problems were more worried, showed more learning problems or had more conflicts with parents.	NA	Determine the change in the emotional health of adolescents before and during the restrictions imposed by the government due to the covid 19 pandemic.
Segre [19] (2021)	Children and adolescent aged 6 to 14	NA	Changes in eating habits and sleep patterns.Increase in anxiety, fear,mood symptoms and greater ease of anger	Increased use of screens, they miss their friends and the practice of their hobbies.	Determine the impact of the pandemic on students, focusing on habit changes and psychological disturbance.
Al-Rahmneh [21] (2021)	Children aged 5 to 11	NA	Increased boredom, irritability and arguments with family members.	Decrease in physical activity and hours of sleep. Increased use of screens.	Assessment of parents’ perceptions of psychoemotional changes in children during the pandemic compared to before the pandemic
Radwan [22] (2021)	Children and adolescent aged 10 to 18	NA	In children, medium levels of stress and moderate levels of anxiety and depression were observed.And moderate levels of stress and depression and severe levels of anxiety were observed in adolescents.	NA	To determine the level of stress, anxiety and depression in students aged 10 to 18 years.
Mohler-Kuo [23] (2021)	Children and adolescent aged 12–17	NA	Higher prevalence of ADHD-related symptoms, followed by symptoms related to ODD, anxiety and depression.	Internet use was higher among boys than among girls.	To assess the impact of Covid 19 confinement on the prevalence of mental symptoms in children/adolescents and young adults.

**Table 3:** Studies characteristics included in the review

Author	Year		Country / Language	Type of study	Participants		EL
					**Sample size**	**Gender**	
Gómez-Becerra ^[9]^		2020	Spain /Spanish	Cross sectional study	N = 972	M = 54.93%; F = 45.06%	IIa
Liang^[18]^		2020	Italy/English	Cross sectional study	N = 1074	M = 52%; F = 48%	IIa
Erades ^[10]^		2020	Spain / Spanish	Cross sectional study	N = 113	M = 51.8%; F = 48.2%	IIa
Orgilés M ^[11]^		2020	Spain / English	Case-control study	N = 96	M = 42.7%; F = 57.3%	III
Morgül E ^[20]^		2020	UK / English	Cross sectional study	N = 927	NA	IIa
Tamarit A ^[12]^		2020	Spain / English	Cross sectional study	N = 523	M = 63.1%; F = 35.8%	IIa
Ezpeleta L ^[13]^		2020	Spain / English	Cross sectional study	N = 226	M = 48.23%; F = 51.77%	IIa
Magson^[17]^		2021	Australia/English	Longitudinal study	N = 248	M = 49.2%; F = 50.8%	IIa
Tang ^[16]^		2021	China/ English	Cross sectional study	N = 4342	M = 51%; F = 49%	IIa
Orgilés M ^[14]^		2021	Spain, Portugal, Italy / English	Cross sectional study	N = 515	M = 54.2%; F = 45.8%	IIa
Francisco R ^[15]^		2020	Spain, Portugal, Italy / English	Cross sectional study	N = 1480	M = 52.8%; F = 47.2%	IIa
Pizarro Ruiz ^[8]^		2021	Spain / English	Cross sectional study	N = 590	Children M = 48.3%; F = 51.7% Adolescents M = 36.6%; F = 63.4%	IIa
Segre^[19]^		2021	Italy/English	Cross sectional study	N = 82	M = 53.7%;F = 46.3%	IIa
Al-Rhamneh^[21]^		2021	Jordan/English	Cross sectional study	N = 1309	M = 54.7%; F = 45.3%	IIa
Radwan^[22]^		2021	Palestian/English	Cross sectional study	N = 420	M = 32.6%; F = 67.4%	IIa
Mohler-Kuo^[23]^		2021	Switzerland/English	Cross sectional study	N = 1146	M = 49.6%; F = 50.4%	IIa

### Results of individual sources of evidence

The main outcomes were categorized in four parts:

1. Lifestyle changes and psycho-emotional manifestations: general results.
2. Psycho-emotional manifestations according to age differentiation.
3. Effects of confinement from a cross-cultural approach.
4. Strategies for promoting resilience: protective factors of the emotional symptoms.

*1. Lifestyle changes and psycho-emotional manifestations: general results.* Among the most frequent lifestyle changes were the school closure and social isolation [13, 14, 21], two measures innate to the situation of confinement that affected not only learning and education, but also the development of social skills, socialization and avoidance of negative feelings such as stress.

On the other hand, there was a significant increase in the use of screens and new technologies as a tool for telematic socialization, entertainment and maintenance of teaching activities [10, 13, 15, 19–21, 23]. A worsening of intrafamily and interpersonal relationship was also observed, as well as a significant increase in sedentary lifestyles due to a decrease in physical activity and outdoor activities. Sleep patterns were also altered, as well as eating habits [10, 11, 13, 15, 19–21].

Among the most frequent manifestations in children and adolescents as a whole, anxiety, stress and depression are three constants, with presentations varying in degree of intensity. Likewise, behavioral disturbances in relation to peers or family and those related to hyperactivity and restlessness stand out. Emotional manifestations are also common, such as feelings of sadness, frustration, worry and boredom [8–23].

*2. Psycho-emotional manifestations according to age differentiation.* Fourteen articles showed the psycho-emotional manifestations depending on age. Five of them deal together with the psycho-emotional affectation on children and adolescents [8, 9, 16, 19, 22]. The remaining articles either deal exclusively with the child [10, 11, 18, 20, 21] or with adolescents [12, 13, 17, 23]. Children or younger patients are those between 2 and 12 years of age and adolescents as those between 13 and 18 years of age (Table 4).

**Table 4:** Prevalence of psychoemotional problems in Adolescents and Children

Authors	Adolescents				
	Anxiety	Depression	Stress	Emotional/ Mood problems	Conduct / Behavior Problems
^a^ Pizarro-Ruiz [8]	43.0%	34.0%	NA	37.7%	46.0%
Tang [16]	Senior Secundary:39.3%	Senior Secundary:32.2%	Senior Secondary: 25.1%	NA	NA
Gomez-Becerra [9]	NA	NA	NA	Mean Score Emotional Symptoms:1.80	Mean Score Conduct Problems: 3.74
Tamarit[12]	Mild:5.5% Moderate:15.1% Severe:5.5% Extremely severe:10.9%	Mild:11.3% Moderate:13.6% Severe:5.9% Extremely severe:7.8%	Mild:11.3% Moderate:12.4% Severe:9.0% Extremely severe:4.0%	NA	NA
Magson [17]	Mean Scores T1 4.6 T2 5.1	Mean Scores T1 3.81 T2 6.12	NA	NA	Mean Scores Conflict-mother T2:2.95 Conflict-father T2:3.04
Ezpeleta [13]	NA	NA	NA	Mean Pre covid:1.21 Mean Post covid:0.82	Conduct: Mean Pre covid:0.98 Mean Post covid:1.23 Behavior: Mean Pre covid:1.46 Mean Post covid:2.85
Segre [19]	46.9%	NA	NA	17%	NA
Radwan [22]	Mean Score:17.98	Mean Score:20.76	Mean Score:19.12	NA	NA
^b^ Mohler-Kuo [23]	13.1%	7.1%	NA	NA	ADHD symptoms:23% ODD symptoms:14.6%
**AUTHORS**	**CHILDREN**				
	**Anxiety**	**Depression**	**Stress**	**Emotional / Mood problems**	**Conduct / Behavior Problems**
^a^ Pizarro-Ruiz [8]	33.2%	22.8%	NA	30.2%	46.8%
Tang [16]	Primary:20.7% Junior Secondary:24.7%	Primary:17.3% Junior Secondary:19.2%	Primary:13% Junior Secondary:14.9%	NA	NA
Gomez-Becerra [9]	NA	NA	NA	Mean Score: Emotional Symptoms:3.84	Mean Score: Conduct Problems: 2.58
Orgilés [11]	Mean Scores SSL Group:2.66 Control Group:5.12	NA	NA	Mean Scores SSL Group:1.12 Control Group:2.75	Mean Scores SSL Group:1.68 Control Group:2.43
Erades [10]	NA	NA	NA	Emotional reactions: 69.6% Indifference:24.1% Discouragement: 23.2%	Behavioral reactions: 24.1% Angry:21.4% Aggressiveness: 17.0%
Morgül [20]	NA	NA	NA	Bored:73.8% Sad:43.4% Worried:52.4% Anxious:45.2%	Behavioral problems:26.2% Irritable:57.1% Angry:48.6% Argue:29.7%
Liang [18]	NA	NA	NA	Total Scores Bored:60.5% Sad:38.4% Worried:48.2% Anxious:25.1%	Total Scores Behavioral problems:9.1% Irritable:39.3% Angry:29.1% Argue:30.1%
Segre [19]	53.1%	NA	NA	19%	NA
Al-Rahamneh [21]	NA	NA	NA	Bored:77.5% Sad:43.4% Worried:38.7% Anxious:44.8%	Behavioral problems:25.4% Irritable:66% Angry: 51.8% Argue:60.7%

^*a*^ *Percentage that scored higher than the clinical sample of SENA scales.* ^*b*^ *The results are given in the table for adolescents, since most of the sample is over 12 years old, and the authors give the results of psychoemotional disturbances at the general level. NA: Not Appear; T1: period pre-pandemic; T2: period during the pandemic;SSL:Super Skills for Life program; ADHD: Attention Deficit Hyperactivity Disorder;ODD: Oppositional Defiant Disorder*

#### Adolescent population

In this population we found higher levels of stress, depression and anxiety [8, 12, 13, 16, 17, 19, 22, 23] with girls having higher scores on these disorders [8, 12, 17, 22], and boys having more family conflicts during confinement [17]. In the study carried out by Tamarit et al. [12] 11.3% of the adolescents presented average levels of stress and depression, 15.1% moderate levels of anxiety, 9% severe levels of stress and 10.9% extremely high levels of anxiety. Although moderate levels of depression, anxiety and stress are the most frequently reported (13.6%,15.1% and 12.4% respectively). While Ezpeleta et al. [13], in a study conducted on a total of 226 adolescents, found a prevalence of stress of 50% and 33% for anxious symptoms. Radwan et al [22] observed higher stress, anxiety and depression scores in the population aged 15–18 years than in the age group 10–14 years, obtaining mean stress, anxiety and depression values of 20.76, 17.98 and 19.12 respectively, with moderate levels in stress and depression and severe levels in anxiety. On the other hand, Mohler -Kuo et al [23] observed a higher prevalence of depression and anxiety in girls than in boys (9.7% vs. 4.6% for depression and 13.6% vs. 12.5% for anxiety). Although the highest prevalences were observed for ADHD (Attention Deficit Hyperactivity Disorder) symptoms (23%), followed by ODD (Oppositional defiant disorder) symptoms (14.6%). Moreover some authors relate these symptoms to the type of housing or information they receive from television or the Internet [12], to not seeing their friends [17] or to fear [9].

Generally, the externalization of psychoemotional distress occurs through sleep disorders with a tendency to insomnia and nightmares, weight changes and eating disorders, as well as emotional manifestations, such as irritability with those closest to them, mood alterations that lead to an increase in intrafamily arguments, sadness, loneliness or isolation, among other manifestations [8, 9, 13, 19].

#### Child population

In children under 13 years of age, the most frequent psychological responses related to behavior were irritability, arguments with the rest of the family or rebellious behavior. [8–11, 18, 20, 21]. Cognitive changes such as difficulty concentrating [11, 18, 19, 21] and increased in anxiety symptoms such as worry, fear or nervousness were also observed [8, 10, 11, 16, 18–21]. Other psychological responses were mood disturbances, such as sadness or feelings of loneliness; sleep disturbances; depression etc.

Morgül et al.[20] conducted a study in children aged 5 to 11 years, observing that the most frequent symptoms were boredom (73.8%) followed by loneliness (64.5%), frustration (61.4%) and irritability (57.1%). While the study by Liang et al. [18], which compared the population of northern and central Italy, showed that the child population of the northern zone presented greater psychological responses than the central zone. Among the most frequent psychological responses were high levels of worry (57%), more feelings of loneliness (46.5%), fear of COVID infection (46.1%), sadness (43.7%) or irritability (41.3%). In the study by Erades et al.[10], the most frequent reactions were emotional (69.6%), followed by sleep disturbances (31.3%) and behavioral disturbances (24.1%). Al-Rahamneh et al. [21] assessed parents’ perceived changes in emotional and behavioral symptoms in children aged 5 to 11 years during the pandemic, and reported that boredom had increased by 77.5%, irritability by 66%, and arguments with family by 60.7%. And in the study of Radwan et al. [22] the mean values of anxiety, depression and stress in the age group 10–14 years were 17.60,14.04 and 14.84 respectively, with medium levels for stress and moderate levels for anxiety and depression.

Finally, Orgilés et al.[11] studied the psychological impact on children who had gone through the Super Skills for Life Program and compared their psychological impact with a control group, observing that the children who had gone through the program had less anxiety, better mood and fewer cognitive alterations.

*3. Effects of confinement from a cross-cultural approach.* Two articles addressed their evaluation cross-culturally by focusing on young people from three different countries: Spain, Italy and Portugal. These studies have been carried out at different times of confinement. The first is at the early stages [15] and the second at a more advanced stage [14] (Table 5).

**Table 5:** Prevalence of psycho-emotional problems in Italy, Spain and Portugal

	ITALY				SPAIN				PORTUGAL			
	Anxiety	Mood		Behavioral	Anxiety	Mood		Behavioral	Anxiety	Mood		Behavioral
Francisco [15]	Anxious:20.5% Nevous:34.1% Mean:2.38	Sad:26.5% Bored:53.8% Mean:1.62		Irritable:36.5% Angry:22.1% Mean:1.38	Anxious:15.7% Nevous:44.3% Mean:2.62	Sad:17.9% Bored:49.4% Mean:1.50		Irritable:43.2% Angry:32.3% Mean:1.82	Anxious:35.9% Nevous:32.3% Mean:2.58	Sad:25.2% Bored:52.2% Mean:1.70		Irritable:45.1% Angry:27.3% Mean:1.68
	**Anxiety**		**Depression**		**Anxiety**		**Depression**		**Anxiety**		**Depression**	
Orgilés [14]	34.1% Mean score:6.60		19.8% Mean score:6.29		56% Mean score:8.69		26.4% Mean score:7.37		26.5% Mean score:5.94		8.5% Mean score:4.74	

In the initial phase of the pandemic, Francisco et al.[15] observed that Italian children had the lowest levels of anxiety and less nutritional, cognitive and sleep disorders than Spanish or Portuguese children. While Portuguese children had more mood disturbances and Spanish children had morebehavioral disturbances.

Regarding anxiety symptoms, Italian children were the most likely to ask about death (14.3%), while Spanish children were more restless (45.5%), more nervous (44.3%), more worried when a parent left home (30.2%), or showed more physical complaints (20.2%). However, Portuguese children were the most uneasy (45.7%), the most worried (44.8%), the most fearful of covid infection (41.2%), the most anxious (35.9%) and the most easily alarmed (22.6%).

Concerning mood, the most prevalent symptoms in Italian children were boredom (53.8%), followed by reticence (27.0%) and sadness (26.5%). Cries was more prevalent in Spanish children (22.7%) and feelings of loneliness (39.5%) and frustration (34.1%) were more prevalent in Portuguese children. They also found that the children who had more sleep problems, more behavioral disturbances and more cognitive disturbances were Spanish children. Thus, in relation to sleep disturbances, they had more difficulty falling asleep (24.3%), more fear of sleeping alone (23.9%), woke up more frequently (15.8%) or had more nightmares (14.8%). The most frequent behavioral symptoms were frequent arguments with other family members (40.4%), incresed dependence on parents (36.4%), more frequent anger (32.3%) and more behavioral problems (29.7%). In addition, Spanish children were more indecisive (16%) and had more concentration problems (30.85%) than Italian or Portuguese children.

Another aspect they studied was feeding problems. In general, they observed that children increased their intake, with the Portuguese increasing their intake the most (27.6%) compared to the Spanish (25.1%) and the Italians (19.9%).

Orgilés et al.[14] studied the presence of anxiety and depression in children and adolescents in Spain, Italy and Portugal. Data were collected 7 weeks after the start of confinement. Of the total population studied, they observed that 38.1% presented anxiety and 19.0% presented depression. Higher levels of psychoemotional discomfort were observed in Italian and Spanish youth, in contrast to Portuguese youth, with Spanish and Italian youth showing higher levels of anxiety and depression compared to Portuguese youth. They found that 56% of Spanish youths were anxiety, compared to 34.1% of Italians youths and 26.5% of Portuguese youths. Similarly, depression values of 26.5% were observed for the Spanish sample, compared to 19.8% in Italians and 8.5% in Portuguese.

*4. Strategies for promoting resilience: protective factors of the emotional symptoms.* Six articles were found that address the different measures that can be taken to promote resilience in a confinement situation [10–12, 16–18].

Orgiles et al.[11] observed that children with more psychological disorders were those who used an emotion-oriented coping strategy. While, in the study by Liang et al. [17] the most commonly used coping strategies were task-focused and emotion focused. Acceptance (62.2%), the seeking affection from others (36.4%) and the highlighting the adventages of being at home (35.6%) were the most commonly used strategies.

Among the different strategies were positive parent-child communication and parenting techniques based on positive reinforcement and the use of time for family tasks and activities, including the reinforcement of a sense of duty and leisure [16].

Another strategy was instruction in healthy lifestyle habits which should include protective measures to avoid contagion by SARS-CoV-2 infection (hand washing, use of masks, social distancing), as well as healthy exercise, sleep and eating habits, maintaining schedules formed under family consensus, to avoid periods of boredom that lead to unhealthy habits [10]. Similarly, promoting communication and relationships with members outside the family unit (social connection), and making use of tradicional media exposure about COVID 19 were also effective [17]. Finally, housing influenced the emotional state of the population studied, was also observed. Thus, those who lived in houses with a balcony or garden showed fewer emotional symptoms [12].

Furthermore, Orgiles et al. [11] demonstrated that the implementation of programs focused on the development of coping strategies and support for young people (such as the Super Skills for Life Program) were also effective in reducing negative psychoemotional manifestations in high-stress situations such as a pandemic, by providing the necessary tools to establish effective coping methods to deal with stressful situations in daily life.

## Discussion

This systematized review of the bibliography has been motivated by the purpose of knowing what has happened at the psychoemotional level with the group of young people and adolescents during the period of confinement in the COVID-19 pandemic.

*1. Psychoemotional manifestations and lifestyle changes.* As indicated by Paricio del Castillo [24], natural disasters, such as epidemics, have consequences at the psychological level since they will cause a threat to life as well as a change in daily routines. This gives rise to post-traumatic stress disorders, bereavement for the loss of loved ones and an increase in psycho-emotional disorders such as increased stress, anxiety and/or depression. Among the most frequent social changes that we find due to an event of this type, are the closure of schools and leisure spaces, social isolation and a decrease in physical activity. All these changes are not only detrimental to physical health, but also have repercussions on the cognitive, behavioral and emotional levels of the population, especially the youngest, both children and adolescents.

In this sense, the articles included in this review show psychoemotional manifestations and life changes in accordance with the article by Paricio del Castillo et al. [24]. With regard to life changes, these were imposed by the governments of each country depending on the emergency situation that was established, giving rise to measures that will irremediably change the daily life of the population. As a consequence of all this, different alterations appear, not only at an emotional level, but also in behavior and conduct in the general population and especially in the younger ones [8–21].

The appearance of psychoemotional alterations in the period of confinement and pandemic of COVID 19 were explained by some authors as follows. Thus, Ezpeleta et al. [13] associated the living conditions imposed by the pandemic with the alterations that occurred at the psychoemotional level. For instance, emotional problems were mainly associated with sleep or health problems and behavioral problems with worsening family relationships or lack of communication with friends. Meanwhile, Gómez-Becerra et al. [9] associated the presence of psychoemotional symptoms with fear. Thus, the emotional symptoms were due to the fear of being infected by the coronavirus and the impact that this fear had on the child. And the behavioral problems were related to behaviors associated with fears about the virus.

*2. Psychoemotional manifestations according to age differentiation.* The different manifestations seen among adolescents and children can be explained by the degree of maturity and independence, not only at the family level, but also in obtaining information from the different media.

### Adolescent population

The results obtained show an elevated prevalence of psychoemotional disorders compared to the period before the Covid-19 pandemic. In 2017, 9% of the population aged 11 to 16 in the United Kingdom had emotional problems and 6.2% had behavioral disorders. While 13% of the population aged 17 to 19 years presented anxiety and 4.8% depression [25]. Likewise, the 2012 study conducted by Besterra [26] in minors observed that 8.5% had emotional problems, 6.7% had behavioral problems, 10.2% had hyperactivity and 7.7% had peer relationship problems. These findings, when compared with the results obtained in this review, are indicative of the extent to which the pandemic and confinement had an impact on the psychoemotional disorders of this population.

A greater manifestation of anxious-depressive and stressful behaviors was found in this group compared to the younger ones. These findings are understood from their psychoemotional maturity and reasoning capacity, as they are derived from their level of awareness of the environment and the world around them, as well as the obligations and responsibilities they have [3, 8, 12, 16, 17].

In addition, adolescents have had less restricted access to unfiltered information about the disease and the overall situation, as well as a higher level of awareness of the family and overall situation in relation to the negative repercussions of contingency measures [14]. On the other hand, they are a group that has its own concerns in relation to studies and relationships with others, which translates into anxious-depressive manifestations with varying degrees of presentation and high levels of stress in the face of uncertainty and forced isolation that prevents them from maintaining friendships and normal daily activities [12, 13, 22].

#### Child population

Regarding the child population, they lack the same degree of responsibility and awareness as adolescents; therefore, their manifestations are not so much linked to awareness of the environment, but rather to what is related to their immediate surroundings and their life routines [8, 10, 11, 20]. Thus, hyperactive or lack of concentration is more common, due to not being able to exhaust the excess energy in relation to the prohibition of outdoor activities [9, 10]. Similarly, emotional and behavioral manifestations were observed, such as leaving tasks unfinished, expressions of disinterest and discouragement, restlessness, anger or irritability. Although the hours of sleep were increased, alterations in the sleep pattern were also observed related to the increased use of new technologies, which could be detrimental to the health of the youngest [8, 10, 11, 18–21]. In addition, at this stage of life, there isa constant search for attention and attachment to parental figures, which increased during confinement, where children became more dependent on their parents as a method of obtaining attention and as a way to alleviate boredom [18].

As in the adolescent population, if the results of the review are compared with the prevalence of psychoemotional disorders before the pandemic, there is a considerable increase in these disorders is observed. In 2017, in the UK, 2.5% of children aged 2 to 4 years had conduct disorders and in children aged 5 to 10 years 5% had conduct disorders and 4.1% had emotional disorders [25]. In the study conducted by Fernández et al. they observed that in the 0 to 5 year-old group, 7.6% had conduct disorders, 17.3% had depressive disorders, 7.7% had anxiety disorders, 5.8% had eating disorders and 1.9% had sleep disorders. These prevalences for the age group 6 to 9 years were 15.4%, 11.5%, 13.3%, 0.7% and 8.1% respectively, while in the age group 10 to 13 years they were 28.6%, 14.4%, 17.4%, 0.8% and 4% respectively [27].

*3. Effects of confinement from a cross-cultural approach*. In order to understand the effects from a cross-cultural level, it is necessary to take into account the differentiation of the measures adopted in the different countries studied. While Spain and Italy adopted restrictive measures, Portugal adopted recommendatory measures.

The differences between the three countries also vary according to the time of observation; thus, the study by Francisco et al.[15] carried out in the early phase of the quarantine, observed a lower impact at the level of externalization of psychoemotional manifestations in Italian youths compared to Spanish and Portuguese youths, even though their measures were among the most restrictive among the three countries. One of the reasons proposed was the adaptation time that each country had had in relation to the time of data collection, since in Italy the measures were implemented earlier than in the other countries studied. Similarly, home characteristics are proposed as a possible explanatory variable for these results, as Italian youth had greater access to the outdoors and relative socialization with the neighborhood because most of the houses had a garden or open space.

On the other hand, in the study carried out by Orgilés et al.[14] in these three countries, at 7 weeks after confinement, different from those obtained previously in the cross-cultural comparison were observed. This study reflects a greater impact on Spanish and Italian youth than on Portuguese youth, due to the degree of severity of the measures implemented in each country, with the most severe measures in Spain and Italy, and a recommendation in Portugal as a way of appealing to individual responsibility.

*4. Strategies for promoting resilience*. There are different measures to promote resilience. The most common were those focused on the parental environment since the family unit spent a lot of time together in a limited space. As shown from the articles included in this review, the measures adopted and which seemed to be the most effective were communication between parents and children, avoiding boredom by carrying out tasks and activities as a family or carrying out activities that include healthy lifestyle habits [10, 11, 16, 18].

There are other measures that are not included in any of the articles, for example, the involvement of health systems to access therapies or consultations with professionals telematically, as indicated in the articles by Paricio del Castillo et al.[24] and Singh et al.[28]. These measures could be used by parents to acquire useful resources that will allow them not only to help their children in developing resilience, but also to help them overcome their own difficulties.

The development of programs to help the population overcome psycho-emotional disturbances could also be another measure that should be included as a strategy. In this regard, the work carried out by Orgiles et al. [11] showed that such therapies would be effective in reducing the frequency of the occurrence of psychoemotional disturbances and therefore a strategy to promote resilience.

Despite the methodology used, the systematized review may have some limitations, such as the small number of studies dealing with the subject. Although at first the initial search returned more results, the established criteria have eliminated part of them. Also, the exclusion of studies that were not in English and Spanish represents a limitation since it does not allow us to know the existence of other articles in different languages that may contain relevant information on the subject to be discussed. In addition, the results have been reported by parents,and not directly by the study population, the children or adolescents themselves, obtaining the parents’ perceptions of their children. Another limitation to take into account is that a meta-analysis has not been carried out, due to the disparity of the data provided by the different studies included in the review.

In spite of these limitations, we consider that the subject is of great importance because, as professionals, we should be aware of the psychoemotional alterations that may occur in the study population in times of stress. In this way, the professional will be able to carry out an appropriate approach with strategies that promote resilience according to the age and type of alteration shown by the patient.

## Conclusions

The presence of psychoemotional manifestations has been present in young people in greater proportion than in the moments prior to confinement, causing a deterioration in psychoemotional quality. As we have already seen, the type of manifestations is determined by the age of the individuals and their development and maturity with respect to the environment. Differences have also been observed depending on the time of observation, as well as between young people from different countries.

Understanding the different spheres of young people and adolescents, their different responses to stressful situations and their adaptive capacities is a key point for the work from the health care point of view and, therefore, from a nursing point of view.

Physical health is an area about which knowledge is extensive, and about which we are most familiar to deal with, however, it is in situations of high stress such as a pandemic, as well as in potentially stressful situations of daily life, when special attention must be paid to the psycho-emotional domain for the preservation of mental and total integrity.

Knowing the impact that confinement has had on the study population will allow us to identify young people who need specialized help and thus be able to intervene before there are irremediable repercussions or long-term effects. As nurses, the manifestations of each age group, as well as the coping strategies they use most are important to know. The purpose of this knowledge will be to be able to influence them to enhance those aspects that are considered beneficial for the promotion of effective resilience. Furthermore, taking into account that health care in the psycho-emotional area is as important as physical health, in the event that nursing competencies do not fully cover the needs of patients, it will allow us to be a liaison between the different levels and health professionals.

Likewise, extrapolating these conclusions as an initiative in daily practice, without the need for a natural catastrophe to occur in order to take into account the mental health of young people is also important.The holistic assessment and intervention is something that should be implemented empirically not only in the theoretical field but also in the practical-clinical development of nursing.

In summary, we can conclude that:

- The type of psico-emotional manifestations in child and adolescent is determined by the age of the individuals and their development and maturity with respect to the environment.
- Knowing the impact that confinement has had on the study population will allow us to identify young people who need specialized help and thus be able to intervene before there are irremediable repercussions or long-term effects.
- Psychoemotional health care is as important as physical health care, and as nurses we can serve as a liaison between the different levels and healthcare professionals in the event that we are unable to fully meet patients’ needs.
- Therefore, the holistic assessment and intervention is something that should be implemented empirically not only in the theoretical field but also in the practical-clinical development of nursing.

## Data Availability

All data generated or analyzed during this study are included in this.

## List of abbrevitions

AHRQ: Agency for Healthcare Research Quality
EL: Evidence Level
NA: Not Appear
PRISMA: Preferred Reporting Items for Systematic Reviews and Meta Analyses
SSL: Super Skills for Life
